# Supplementary material for: Cytoskeletal stiffening in synthetic hydrogels
Source: Nat Commun. 2019 Feb 5;10:609. doi: 10.1038/s41467-019-08569-4 (PMC6363731; doi:10.1038/s41467-019-08569-4)
Supplement: Supplementary file 1 — Supplementary Information [file 41467_2019_8569_MOESM1_ESM.pdf]

Supplementary Information

Cytoskeletal stiffening in synthetic hydrogels

Paula de Almeida et al.

Supplementary Methods

Synthesis of dibenzocyclooctyne-functionalized acrylamide. The dibenzocyclooctyne-amine (12 mg, 0.044 mmol, Click Chemistry Tools) was dissolved in dry THF (10 mL) and acryloyl chloride (13.4 µL, 0.13 mmol, Sigma Aldrich) with a drop of trimethylamine (JT Baker). The reaction mixture was stirred for two hours at room temperature after which the mixture was poured into ice water (50 mL). The aqueous mixture was extracted with dichloromethane (3 × 50 mL). The combined organic layers were washed with a 0.1 M HCl solution, dried with $MgSO_4$ and the solvent was evaporated *in vacuo*. The resulting product was purified by precipitation in *n*-hexane and vacuum dried to yield 4.4 mg (30 %) as a yellow solid. ^{1}H-NMR (Bruker Avance III, 400 MHz, $CDCl_3$): δ 7.67 (d, J = 7.4 Hz, 1H), 7.38 (m, 7H), 6.28 (s, 1H), 6.10 (m, 1H), 5.92 (m, 1H), 5.53 (d, J = 1.4 Hz, 1H,), 5.16 (d, J = 13.9 Hz, 1H,), 3.72 (m, H,), 3.46 (m, 1H,), 3.29 (m, 1H,), 2.56 (m, 1H), 2.00 (m, 1H,). ^{13}C NMR (101 MHz, $CDCl_3$: δ 170.09, 164.43, 151.55, 148.35, 132.38, 131.58, 129.53, 128.95, 128.25, 128.07, 127.73, 126.83, 124.92, 122.46, 121.44, 114.29, 108.02, 54.86, 48.60, 35.04, 34.12. FT-IR (ATR, cm^{-1}) 3308, 2925, 1658, 1448, 1400, 1230, 754. MS (m/z): $[M]^+$ calcd. for $C_{21}H_{18}N_2O_2$,330.4; found 331.1.

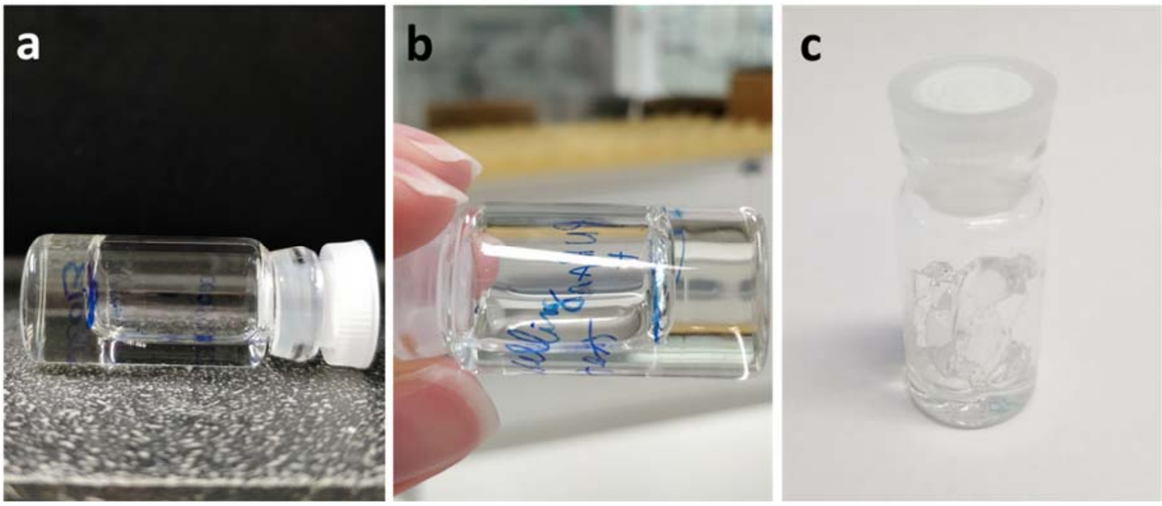

Supplementary Figure 1 | Swelling behaviour of the gels. (a) PIC (3 mg/mL) and (**b**) PIC/PNIPAM (3/40 mg mL^{-1}) do not present visible swelling after 24 hours in contact with water, marked with the constant volume in comparison to the original gel (blue line). (**c**) The swelling of PNIPAM single-network (40 mg mL^{-1}) in water is quite intense, causing the breakage of the gel.

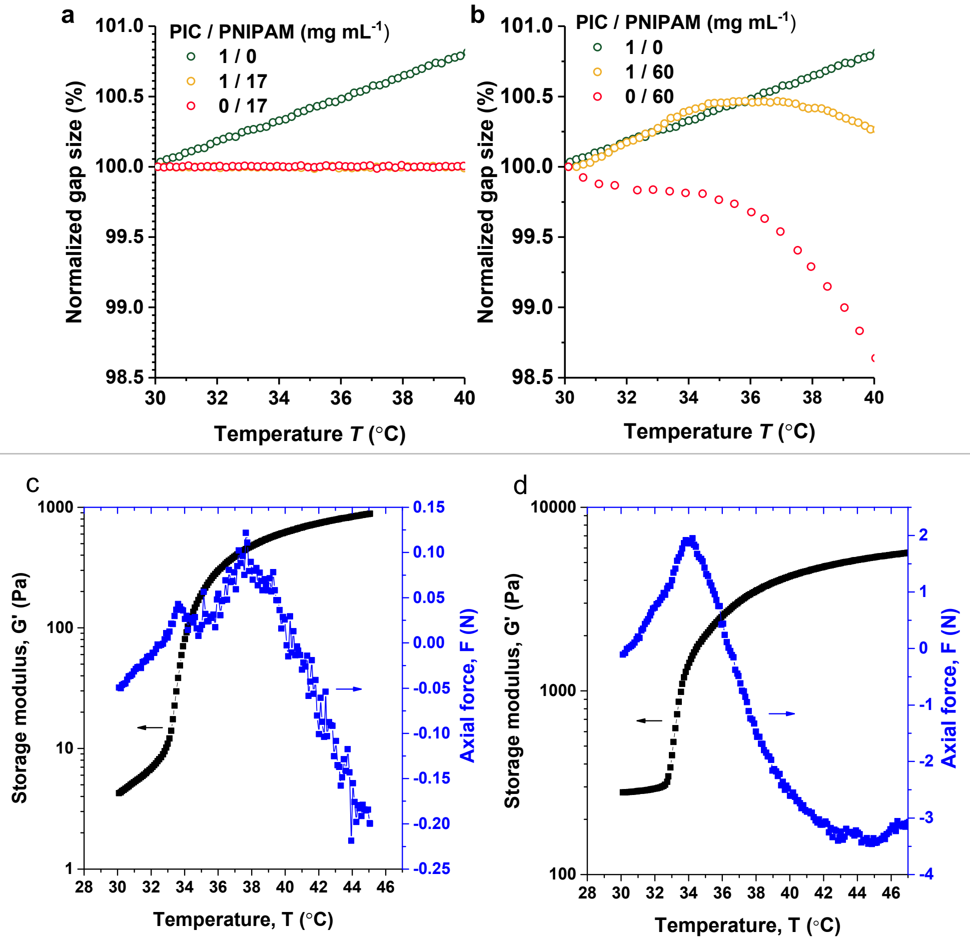

Supplementary Figure 2 | Variation in gap size and axial force with temperature. (**a**,**b**) The development of normal force due to the PNIPAM collapse causes changes in the gap size for (**a**) PIC/PNIPAM 1/17 (**b**) 1/60 mg mL^{-1} and equivalents single-networks. The gap size does not change significantly with temperature, except for the single-network PNIPAM 60 mg mL^{-1}. PIC only gel presents a small expansion with temperature. The presence of PIC network prevents the shrinkage of the hybrid gel and a small change in size is observed. To ensure good contact between the rheometer plates and the soft sample, we record data under axial force control. When the normal force exceeds |0.2| N, the gap size changes. This means that the gaps size is a direct measure for the formation of normal stresses in our samples. (**c**,**d**) Axial force variation for the samples **(c)** PIC/PNIPAM 1/17 and **(d)** 1/60 mg mL^{-1}. Both samples show a negative (pull down) force with PNIPAM collapse, right after the development of a positive axial force. Note that the axial force scale for the higher PNIPAM concentration is much larger than for the unpercolated network.

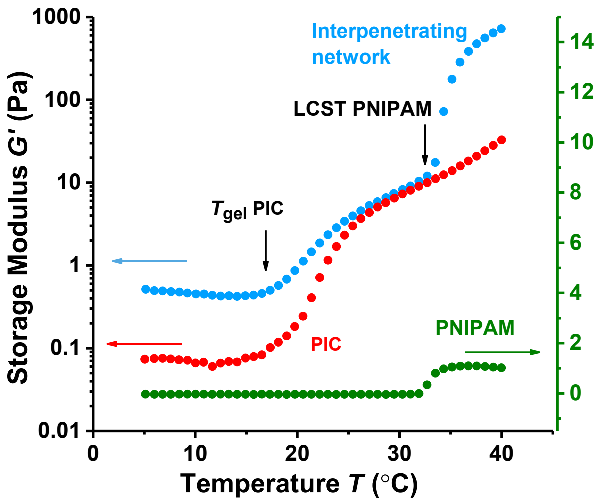

Supplementary Figure 3 | Stiffening effect. Mechanical properties of the IPN PIC/PNIPAM (1/17 mg mL^{-1}) with the PNIPAM data on a linear scale.

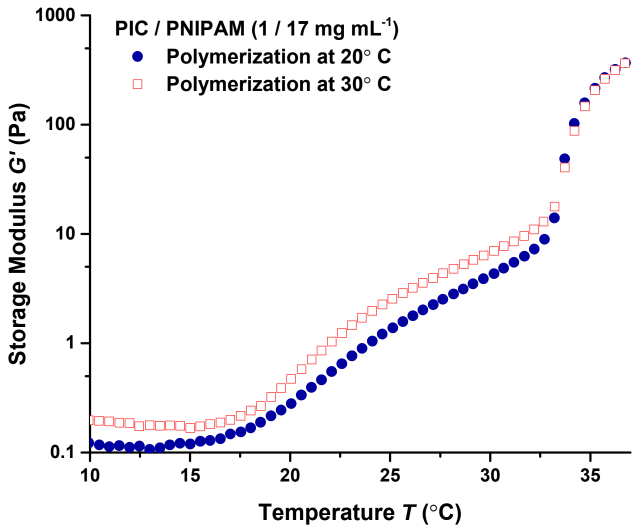

Supplementary Figure 4 | Influence of polymerisation temperature. Storage modulus as a function of temperature for PIC/PNIPAM double network hydrogels formed at T = 20 (blue circles) and 30 °C (red squares).

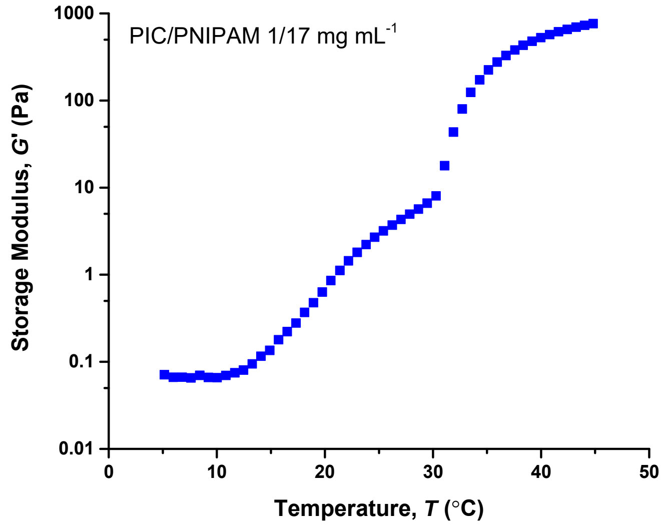

Supplementary Figure 5 | Stiffening in serum-free media. Storage modulus as a function of temperature for PIC/PNIPAM double-network hydrogel formed in serum-free media. Both T_{gel} of PIC and the LCST of PNIPAM shift a few degrees to lower temperature.

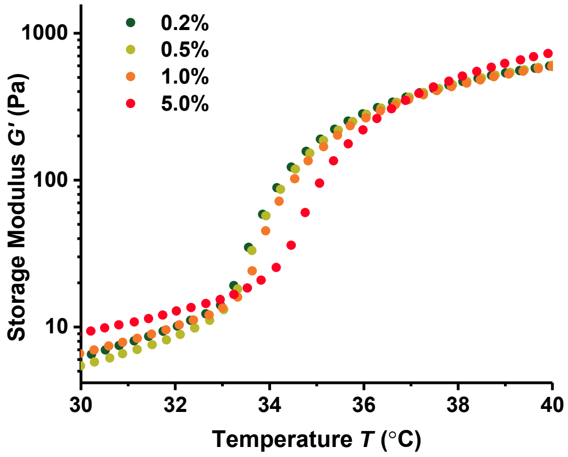

Supplementary Figure 6 | Impact of the PNIPAM crosslinker concentration. The crosslinker concentration c_{MBAA} does not affect the mechanical properties of the hybrid PIC/PNIPAM (1/17 mg mL^{-1}). The crosslinker concentration is expressed in mol-% of NIPAM.

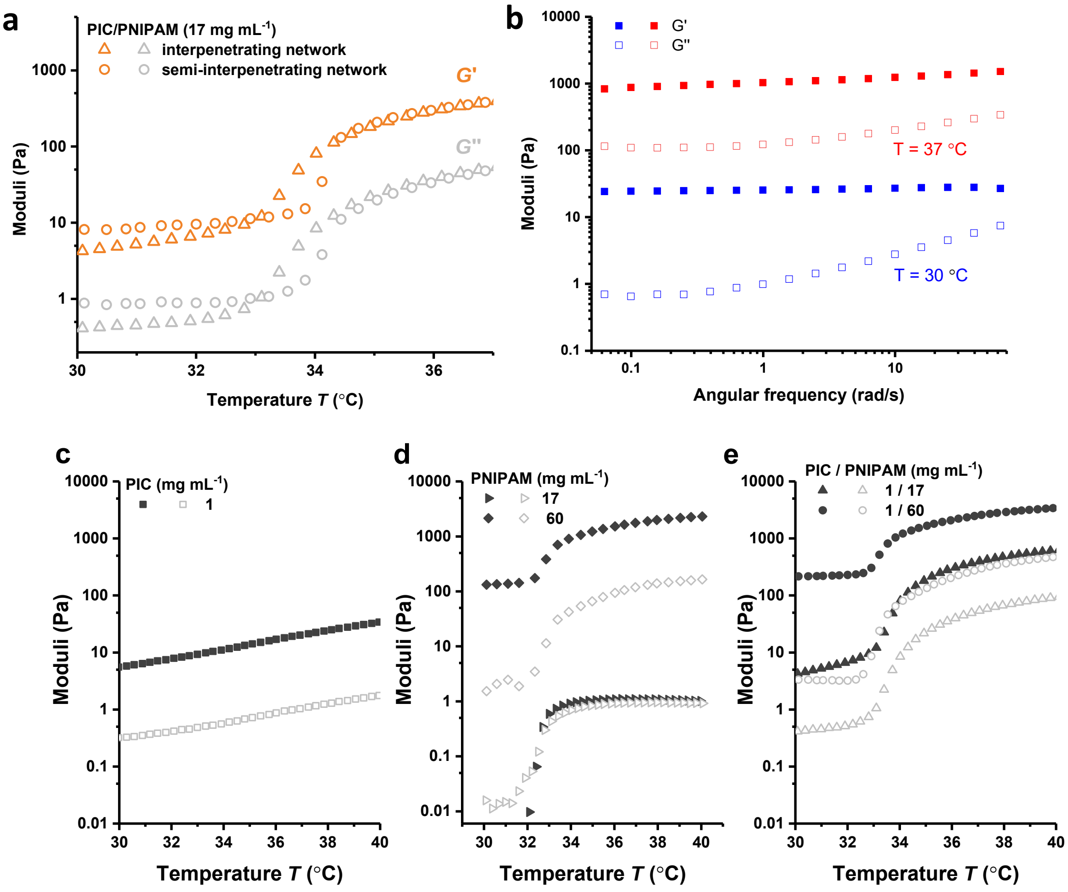

Supplementary Figure 7 | Viscoelastic properties of IPNs and semi-IPNs. (**a**) Storage and loss moduli as a function of temperature for the semi-IPN and IPN if PIC/PNIPAM (1/17 mg mL^{-1}). The hydrogels behave as viscoelastic materials with G' >> G''. (**b**) Storage and loss moduli for the PIC/PNIPAM 1/40 at temperatures below (T=30 °C) and above (T=37 °C) PNIPAM LCST. The loss modulus presents a small dependence with frequency. (**c**-**e**) Storage and loss moduli as a function of temperature for PIC only gels (**c**) PNIPAM-only gels (**d**) and PIC/PNIPAM IPNs (**e**) at different PNIPAM concentrations.

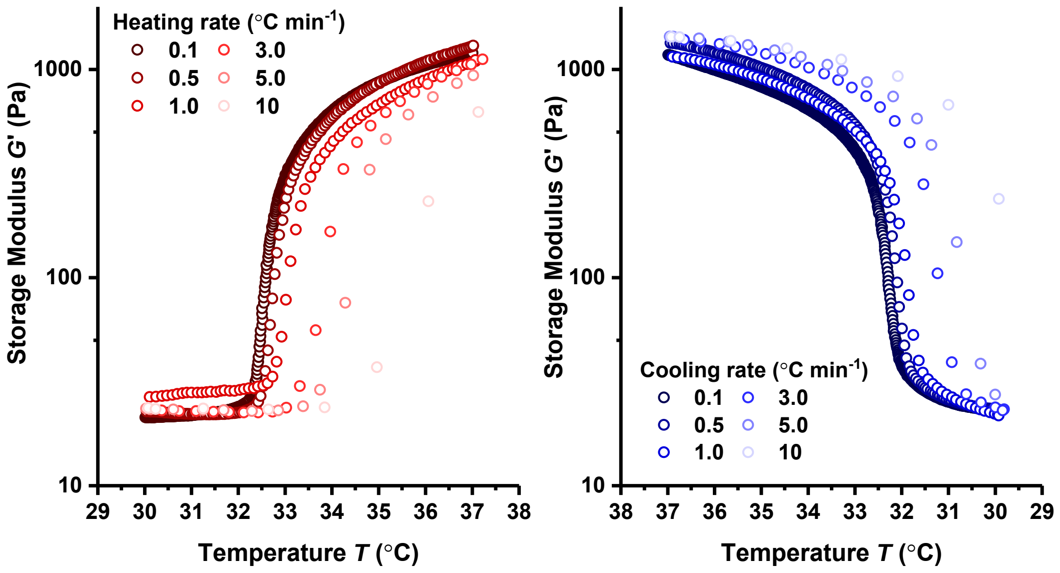

Supplementary Figure 8 | Stiffening rates as a function of heating and cooling rates. Effect of the heating/cooling rate to the stiffening effect (PIC/PNIPAM 1/40 mg mL^{-1}). Up to 3 °C min^{-1} the transition temperature is similar, although the effect is much sharper at slow rates. Faster rates are limited to the heat capacity of the systems, as seen for the late stiffening at rate 10 °C min^{-1}.

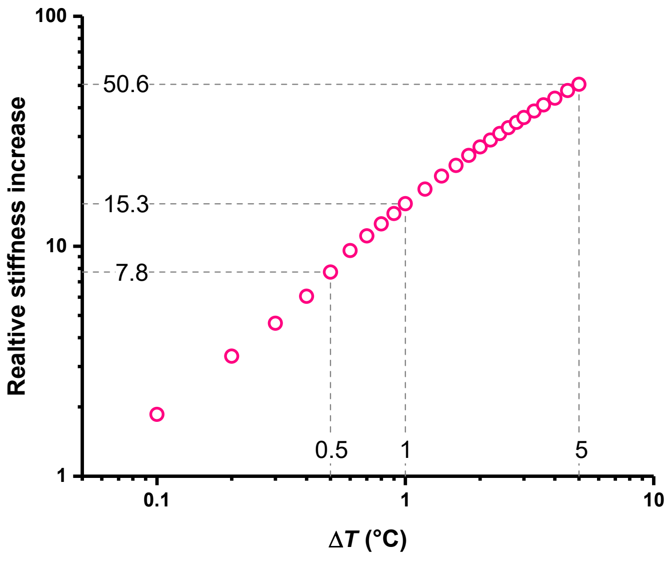

Supplementary Figure 9 | Maximum achievable stiffness jump in a temperature window. Relative stiffness increase, defined as $G'_{T+\Delta T}/G'_{T}$ for a temperature window ΔT, for the hydrogel PIC/PNIPAM 1/17 at a heating rate of 0.1 °C min^{-1}. The hybrid IPN stiffens about 8 fold in 0.5 °C, 15 fold in 1 °C and 51 fold in 5 °C.

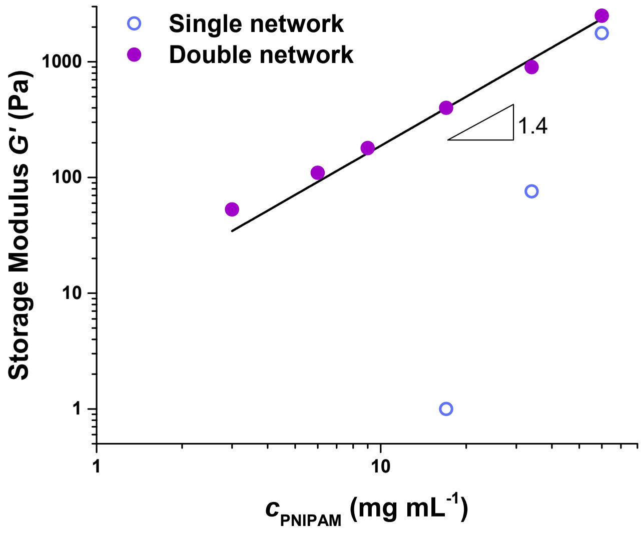

Supplementary Figure 10 | Storage modulus dependence on the PNIPAM concentration. Storage moduli *G′* of the IPNs (c_{PIC} = 1 mg mL^{-1}, purple data) and PNIPAM only networks (open blue) as a function of the PNIPAM concentration beyond the LCST (= 37 °C). The line is a power law fit: $G' \propto c_{PNIPAM}^{1.4}$ for the hybrids. Single networks can only be measured for $c_{PNIPAM} \geq$ 17 mg mL^{-1}. Single and hybrid networks with c_{PNIPAM} = 60 mg mL^{-1} show similar *G′*. Beyond this concentration, the mechanics are dominated by the PNIPAM network.

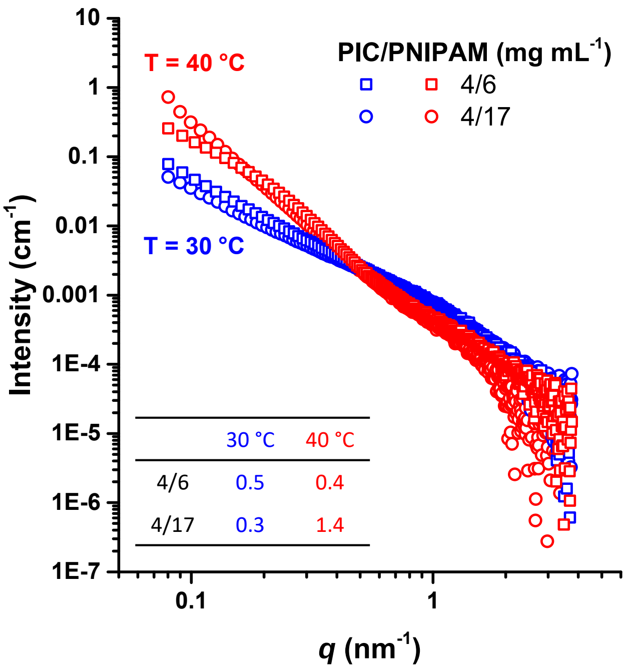

Supplementary Figure 11 | Small angle X-ray scattering of PIC/PNIPAM hybrid hydrogels. SAXS curves with PIC/PNIPAM concentrations of 4/6 (squares) and 4/17 (circles) mg mL^{-1} at T = 30 and 40 °C. The profiles allow a clearer visualization of the large increase in the scattering intensity at low scattering angles q. The fitted I_0 values of the correlation length model are depicted in the inset.

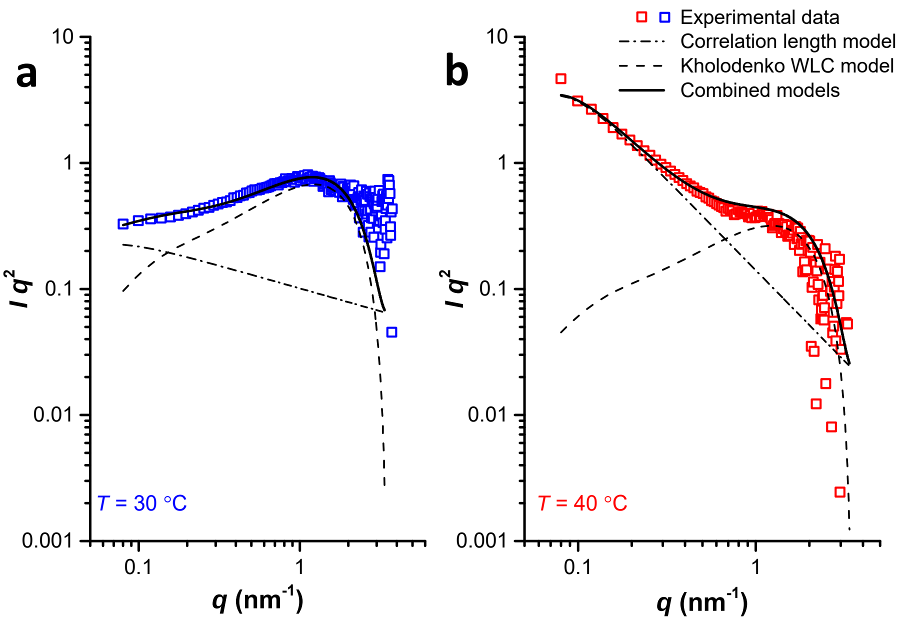

Supplementary Figure 12| Kratky analysis of the scattering data. Iq^2 as a function of q for the PIC/PNIPAM 4/17 mg mL^{-1} at T = 30 °C (**a**) and T = 40 °C (**b**). The individual contribution of the correlation length model (dash-dot line) and Kholodenko worm-like chain model (dashed line) are shown.

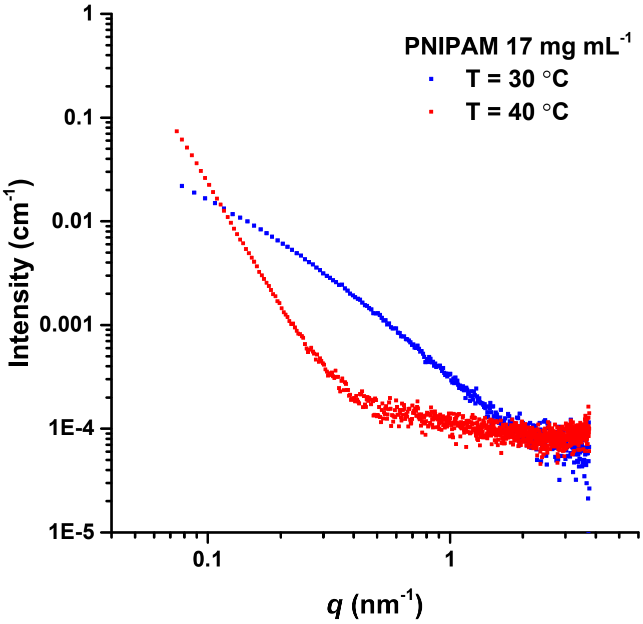

Supplementary Figure 13 | Small angle X-ray scattering of PNIPAM networks. Scattering profile of PNIPAM (17 mg mL^{-1}) at T = 30 °C and T = 40 °C. At low q values, the scattering intensity for the PNIPAM network is much smaller in comparison to the hybrid hydrogels.

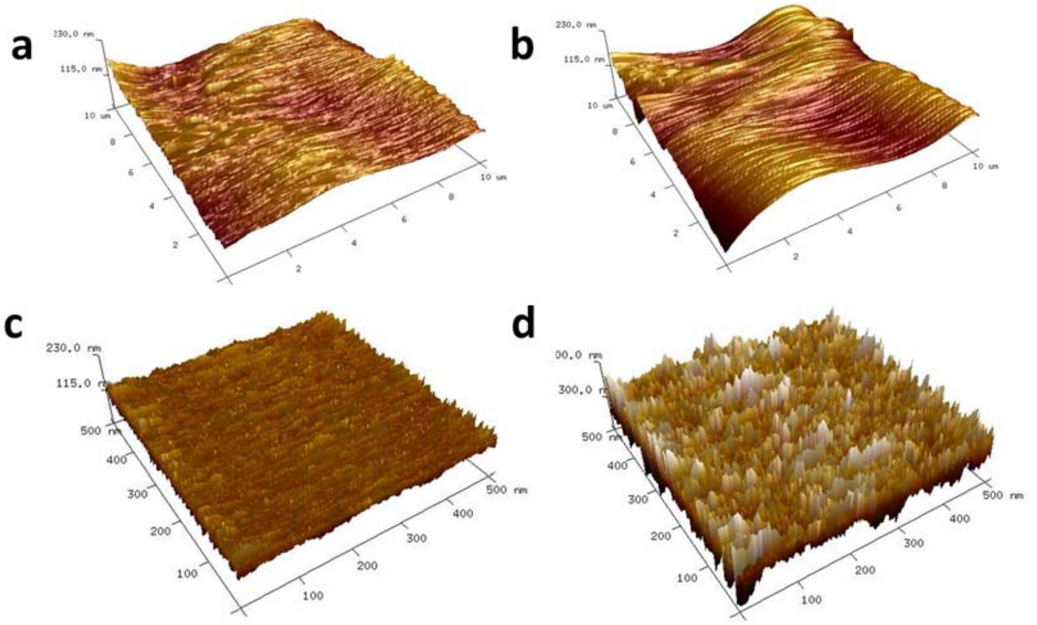

Supplementary Figure 14 | AFM analysis. AFM images for PIC hydrogel at (**a**) 25 °C and (**b**) 37 °C, and for the PIC/PNIPAM hydrogel (4/17 mg mL^{-1}) at (**c**) 25 °C and (**d**) 37 °C. The roughness of PIC hydrogel is almost invariable with temperature, while for the hybrid gel the roughness increases from 12 to 80 nm with LCST (note the change in *z*-scale).
